# Current scenario of peptide-based drugs: the key roles of cationic antitumor and antiviral peptides

**DOI:** 10.3389/fmicb.2013.00321

**Published:** 2013-10-31

**Authors:** Kelly C. L. Mulder, Loiane A. Lima, Vivian J. Miranda, Simoni C. Dias, Octávio L. Franco

**Affiliations:** Programa de Pós-Graduação em Ciências Genômicas e Biotecnologia, Centro de Análises Proteômicas e Bioquímicas, Universidade Católica de BrasíliaBrasília, Brazil

**Keywords:** cationic peptides, antiviral, antitumor, target selectivity, therapeutic drugs

## Abstract

Cationic antimicrobial peptides (AMPs) and host defense peptides (HDPs) show vast potential as peptide-based drugs. Great effort has been made in order to exploit their mechanisms of action, aiming to identify their targets as well as to enhance their activity and bioavailability. In this review, we will focus on both naturally occurring and designed antiviral and antitumor cationic peptides, including those here called promiscuous, in which multiple targets are associated with a single peptide structure. Emphasis will be given to their biochemical features, selectivity against extra targets, and molecular mechanisms. Peptides which possess antitumor activity against different cancer cell lines will be discussed, as well as peptides which inhibit virus replication, focusing on their applications for human health, animal health and agriculture, and their potential as new therapeutic drugs. Moreover, the current scenario for production and the use of nanotechnology as delivery tool for both classes of cationic peptides, as well as the perspectives on improving them is considered.

## Introduction

Antimicrobial peptides (AMPs) are natural peptides found in microorganisms, plants, and animals, and are considered the evolutionarily conserved effectors in innate immunity (Hancock and Chapple, 1999; Hancock and Sahl, 2006). These peptides can be classified by physical–chemical properties such as cationic, anionic, hydrophilic, and amphipathic (Peter et al., 2010). Although they are diverse in length and sequence, two physical-chemical features are often the hallmarks of these molecules: they are cationic, often ranging from +2 to +7 at pH 7, and amphipathic, therefore their stereogeometry confers relatively polarized hydrophilic and hydrophobic facets (Nguyen et al., 2011). Despite their structural conservation, they have a broad spectrum of activity such as antibacterial (Okubo et al., 2012; Tavares et al., 2012), antioxidative (Power et al., 2013), antihypertensive (Escudero et al., 2012), antifungal (Mandal et al., 2013), antiviral (Findlay et al., 2013), antitumor as well as modulation of the immune response (Silva et al., 2012). The modulation of the immune response role is specific to a group of peptides named host defense peptides (HDPs). Moreover, multiple functions may be associated with a single peptide according to the concept of promiscuity. They may act over different targets, therefore presenting different functions depending on their physical-chemical (namely pure promiscuity) or on their amino acid modification (namely family promiscuity) (Franco, 2011). These features confer to AMPs and HDPs many physiological advantages over other molecules for application in the field of drug development. As is summarized here, many AMPs and HDPs isolated from a wide variety of organisms including mammals, amphibians, insects, plants, and bacteria have been reported to have antiviral and/or antitumor activities. This review comprises their mechanisms of action and targets, as well as their potential as new therapeutic strategies to combat both viruses and malignant cells.

### Cationic antitumor peptides

Cancer has become a major concern in relation to human morbidity and mortality. All types of cancer are characterized by irregular cell growth originating from a small number of inherited or environmentally-stimulated genetic mutations (Renan, 1993). Many strategies have been adopted to combat the propagation of cancer cells and their elevated growth such as chemotherapy, surgery, and radiation (Wang and Zhang, 2013). These typical procedures have often been revealed to be non-specific for cancer cells (Yang et al., 2013), additionally acting on the cell division of healthy cells, consequently impairing the restoration of normal tissues (Smith et al., 2000). Antitumor drugs are subject to differences in absorption, metabolism, and target tissue, which can be particular to each patient; moreover, tumors can be positioned in places into which drug penetration is impaired or possibly sheltered by restricted environments due to amplified hydrostatic pressure in the tissue or modified tumor vasculatures (Szakacs et al., 2006). Furthermore, the intrinsic or acquired drug resistance is considered the widespread cause for tumor recurrence (Szakacs et al., 2006).

Acknowledging the limitations of these currently available therapies, researchers have been encouraged to seek novel anticancer agents with only one exclusive mechanism of action (Szakacs et al., 2006; Hoskin and Ramamoorthy, 2008). In this context, natural AMPs from different sources and their synthetic analogs have been the basis for a number of studies performed to discover new therapies for treating malignant cells (Table 1).

**Table 1:** **Cationic antitumor peptides from different sources, their application, and their mechanisms of action**.

**Peptides**	**Source**	**Group**	**Application**	**Mechanism of action**	**References**
AGAP	*Buthus martensii*	Insect	Lymphoma, leukemia, human malignant glioma, human colon cancer	Cell cycle arrest	Cao et al., 2010; Zhao et al., 2011; Gu et al., 2013
Alloferon	*Calliphora vicina*	Insect	Leukemia	Induces NK and IFN—Immune-modulatory	Chernysh et al., 2002
Aplidine	*Aplidium albicans*	Tunicate	Melanoma, non-small cell lung, prostate, ovarian, colorectal	Multifactorial apoptosis inducer/cell cycle arrest/inhibition of protein synthesis	Faivre et al., 2005
Apratoxin A	*Lyngbya majuscula*	Cyanobacteria	HeLa (human cervical cancer)	Cell cycle arrest	Ma et al., 2006
Arenastatin A	*Dysidia arenaria*	Sponge	Human KB carcinoma	NR	Kobayashi et al., 1994
Aurein 1.2	*Litoria raniformis*	Frog	Leukaemia, lung, colon, CNS, melanoma, ovarian, renal prostate, breast	Barrel stave mechanism	Rozek et al., 2000
BMAP-27	*Bos taurus*	Mammal	Leukemia, human erythromyeloblastoid leukemia, human leukemic monocyte lymphoma	Influx of Ca^2+^	Risso et al., 1998, 2002
BMAP-28				DNA fragmentation Increase membrane permeabilization	
Brevinin-2R	*Rana ridibunda*	Frog	Leukemia, lymphoma, colon carcinomas, fibrosarcoma, breast adenocarcinoma, lung carcinoma	Depolarize the transmembrane potential/lysossomal pathway	Ghavami et al., 2008
Buforin IIb	*Bufo bufo gargarizans*	Frog	Leukemia, breast cancer, non-small cell lung cancer, CNS cancer, melanoma, renal, ovarian, prostate and colon cancer	Apoptosis by a mitochondria-dependent pathway/caspase-9 activation/cytochrome c	Lee et al., 2008
Cecropins	*Hyalophora cecropia*	Insect and mammals	Leukemia, bladder	Carpet mechanism/Membrane disruption	Steiner et al., 1981; Chen et al., 1997; Papo and Shai, 2005; Lehmann et al., 2006; Suttmann et al., 2008; Xu et al., 2008
Cherimolacyclo peptide C	*Annona cherimola*	Plant	Human KB carcinoma	NR	Wele et al., 2004
Citropin 1.1	*Litoria citropa*	Frog	Leukemia, lung, colon, CNS, melanoma,ovarian, renal, prostate, breast	Carpet mechanism	Doyle et al., 2003
*Cn-*AMP1	*Cocos nucifera*	Plant	Colorectal adenocarcinoma	NR	Silva et al., 2012
Coibamide A	*Leptolyngbya sp.*	Cyanobacteria	Breast, CNS, colon, melanoma, leukemia, ovarian	Cell cycle arrest	Medina et al., 2008
CPAP	*Chlorella pyrenoidosa*	Algae	Human liver cancer	Condensation/ fragmentation of nuclear chromatin	Wang and Zhang, 2013
Cr-ACP1 and Cr-AcACP1	*Cycas revoluta*	Plant	Human epidermoid cancer, colon carcinoma	Cell cycle arrest	Mandal et al., 2012
CS5931	*Ciona savignyi*	Tunicate	Human colorectal carcinoma	Mitochondrial pathway of apoptosis	Cheng et al., 2012
Cyclotide	*Clitoria ternatea*	Plant	Lung cancer	NR	Sen et al., 2013
Cycloxazoline	*Lissoclinum bistratum*	Ascidian	MRC5CVl fibroblasts, T24 bladder carcinoma, leukemia	Cell cycle arrest/inhibition of cytokinesis	Hambley et al., 1992; Watters et al., 1994
Dianthins E	*Dianthus superbus*	Plant	Liver hepatocellular cells	NR	Hsieh et al., 2004
Didemnin	*Trididemnun solidum*	Ascidian	Leukemia, melanoma	Protein synthesis inhibition/apoptosis	Rinehart et al., 1983
Dolastatin 10	*Symploca sp.*, *Dolabella auricularia*	Cyanobacteria, mollusk	Murine leukemia cells, lung cancer	Bcl-2 phosphorylation/Caspase-3 protein activation	Bai et al., 1990; Kalemkerian et al., 1999
Gaegurins	*Rana rugosa*	Frog	Kidney, lung, colon, breast, stomach, liver, prostate, skin, ovary	Pore formation by carpet-model	Won et al., 2006
Geodiamolide H	*Geodia corticostylifera*	Sponge	Breast cancer	Altering the actin cytoskeleton	Freitas et al., 2008
Glidobactins A, B and C	*Polyangium brachysporum sp*.	Bacterium	Melanoma, leukemia, colon carcinoma	NR	Oka et al., 1988
Homophymines	*Homophymia sp.*	Sponge	Pancreatic cancer, human erythromyeloblastoid leukemia, breast, liver hepatocellular, human KB carcinoma, human colon adenocarcinoma, human ovarian, human prostate, glioblastoma, lung epithelial cells	NR	Zampella et al., 2009
Human alpha-defensin-1	*Homo sapiens*	Human	Human lung adenocarcinoma	Apoptosis by cytochrome c from mitochondria (mitochondrial pathway)	Xu et al., 2008
Human neutrophil Peptides (HNP-1: β-defensin)	*Homo sapiens*	Human	Leukemia and solid tumor	Induce membrane proteolysis	McKeown et al., 2006
Jamaicamide A	*Lyngbya majuscula*	Cyanobacterium	Human lung, neuroblastoma cell	NR	Edwards et al., 2004
Jaspamides	*Jaspis splendens*	Sponge	Lymphoma	Caspase-3 activation/decreasing in Bcl-2 protein expression	Ebada et al., 2009; Ghosh et al., 2010
Kahalalide F (KF)	*Elysia rufescens*	Mollusk	Colon, breast, non-small cell lung, prostate carcinoma, melanoma, hepatocellular carcinoma	Inhibit expression of genes involved in DNA replication/modifies lysosome membrane/apoptosis inducer	Hamann et al., 1996; Gracia et al., 2006; Singh et al., 2008
Keenamide A	*Pleurobranchus forskalii*	Mollusk	Leukemia, human lung adenocarcinoma, human colon adenocarcinoma	NR	Wesson and Hamann, 1996
Lactoferricin B	*Bos taurus*	Mammal	Human leukemia, fibrosarcoma, carcinoma, neuroblastoma	Mitochondria pathway of apoptosis/cytochrome c release/activation of the caspase cascade	Mader et al., 2005; Eliassen et al., 2006
LL-37	*Homo sapiens*	Human	Ovarian cancer	Pore formation by carpet-model	Chuang et al., 2009
Longicalycinin A	*Dianthus superbus*	Plant	Human liver carcinoma	NR	Hsieh et al., 2005
Lunasin	Soybean and other seeds	Plant	Breast cancer	NR	Hsieh et al., 2010
Lyngbyabellins	*Lyngbya majuscula*	Cyanobacterium	KB carcinoma	NR	Williams et al., 2003
Magainins	*Xenopus laevis*	Frog	Hematopoietic tumor, melanoma, ovarian cancer, bladder cancer, human cervical carcinoma	Mitochondria pathway of apoptosis/Pore formation by toroidal model/cytochrome c release/activation of the caspase cascade/Carpet mechanism	Zasloff, 1987; Cruciani et al., 1991; Jacob and Zasloff, 1994; Takeshima et al., 2003; Lehmann et al., 2006
Malevamide D	*Symploca hydnoides*	Cyanobacterium	Leukemia, lung cancer, human colon carcinoma	NR	Horgen et al., 2002
Melittin	*Apis mellifera*	Insect	Human hepatocellular carcinoma	Influx of Ca^2+^/carpet mechanism/toroidal pore	Tosteson et al., 1985; Wang et al., 2009a
Mere15	*Meretrix meretrix*	Bivalve	Human lung adenocarcinoma	Induce release of cytochrome c/cleavage of caspases/poly ADP-ribose polymerase	Wang et al., 2012
Microcolin A	*Lyngbya majuscule*	Cyanobacterium	Breast carcinoma	Induction of apoptosis	Zhang and Longley, 1999
Mollamide	*Didemnum molle*	Ascidian	Leukemia, human lung carcinoma, human colon carcinoma	NR	Carroll et al., 1994
Pardaxinis	*Pardachirus marmoratus*	Fish	Human sarcoma	Elevation of caspase activities, disruption of the mitochondrial membrane	Huang et al., 2011
Phakellistatin 13	*Phakellia fusca*	Sponge	Human hepatoma	NR	Li et al., 2003
RA-XVII	Rubiaceous plants and *Aster tataricus*	Plant	Leukemia	Activation of caspase activity	Hitotsuyanagi et al., 2004
Sansalvamide A	*Fusarium ssp.*	Fungi	Pancreatic, colon, breast, and prostate sarcoma, melanoma	Cell cycle arrest	Vasko et al., 2010
Scopularide A and B	*Scopulariopsis brevicaulis* and *Tethya Aurantium*	Marine fungi, sponge	Pancreatic tumor, colon tumor	NR	Yu et al., 2008
*St*AP1 and *St*AP3	*Solanum tuberosum*	Plant	Leukemia	Induces apoptosis	Mendieta et al., 2010
Symplostatin 1	*Symploca sp.*	Cyanobacterium	Breast, colon tumor	Disrupts microtubules/mitotic arrest/induces apoptosis	Mooberry et al., 2003
Tachyplesin I	*Tachypleus tridentatus*	Japanese horseshoe crab	Human hepatocellular carcinoma, prostate carcinoma	Non-cytolytic mechanism	Chen et al., 2005
Tamandarins A and B	Family *Didemnidae*	Ascidian	Pancreatic carcinoma, prostatic cancer, head and neck carcinoma	Protein synthesis inhibition	Vervoort et al., 2000
Trapoxins A and B	*Helicoma Ambiens*	Fungi	Colorectal cancer	NR	Itazaki et al., 1990
Virenamides A–C	*Diplosoma virens*	Ascidian	Leukemia, human lung carcinoma, human colon carcinoma	Protein synthesis inhibition	Carroll et al., 1996
Viscotoxins	*Viscum coloratum, Viscum álbum*	Plant	Osteoblast-like Sarcoma, Yoshida sarcoma (rat)	NR	Xu and Jin, 1999
Vitilevuamide	*Didemnum cuculiferum* (Ascidians)	Ascidian	Colon tumor, lung cancer, melanoma, kidney cancer	Tubulin polymerization/cell cycle arrest	Edler et al., 2002

NR, Not reported. KB, nasophryngeal cancer; CNS, central nervous system.

**Figure 1:**
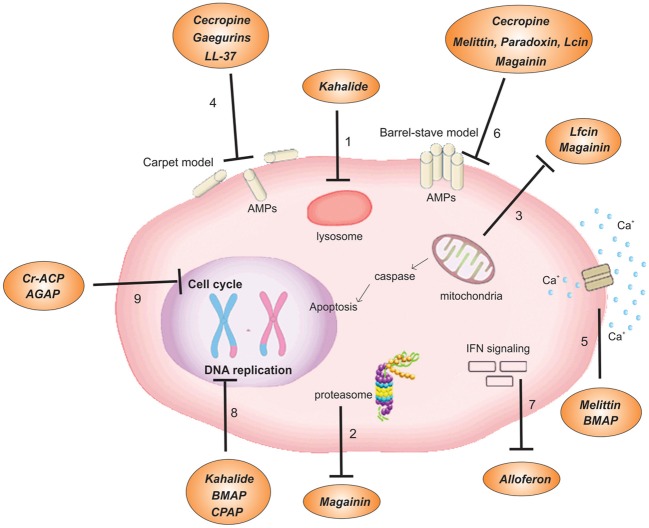
**Mechanisms of action of cationic antitumor peptides**. (1) Modification of the lysosome membrane leading to an acidification of the intracellular environment and cell death. (2) Amplification of the proteasome activity. (3) Induction of mitochondrial pathway of apoptosis by either the cytochrome c release into the cytoplasm or activation of the caspase cascade. (4) Pore formation by the carpet model. (5) Increase of the influx of Ca^2+^. (6) Formation of pore by either the toroidal or the barrel-stave models. (7) Activation of an immune modulatory pathway by induction of NK and IFN. (8) Inhibition of genes involved in DNA replication. (9) Arrest of cell cycle G0, G1, or S phases. Lfcin, lactoferrcin; BMAP, bovine myeloid antimicrobial peptide; Cr-AMP, Chlorella pyrenoidosa antitumor polypeptide.

Cationic antitumor peptides have been suggested as promising agents for antitumor therapy due to their numerous advantages over other chemical agents such as their low molecular masses, relatively simple structures, greater specific cytotoxicity to tumor cells over healthy cells, fewer adverse reactions, ease of absorption, a variety of routes of administration and low risk for inducing multi-drug resistance (Alberts et al., 1980; Mader and Hoskin, 2006; Hoskin and Ramamoorthy, 2008; Schweizer, 2009; Riedl et al., 2011; Liu et al., 2013). They can also function in combination with conventional therapies and other potential anticancer molecules, usually improving the results of therapy (Chuang et al., 2009; Wang et al., 2009a). The intrinsic relationship between their chemical structure, i.e., their cationic and hydrophobic features, and their high specificity to tumor cells is likely to be the key role for their cytotoxicity. These characteristics allow the cationic AMPs to bind to and invade cancer cells, quickly disrupting membranes and leading to the outflow of intracellular contents and consequent cell death (Yeaman and Yount, 2003; Leuschner and Hansel, 2004; Jenssen et al., 2006). This happens due to the negatively-charged tumor cell membrane [derived from a greater than normal expression of anionic molecules such as sialic acid-rich glycoproteins, phosphatidylserine (PS) or heparan sulfate], which present essential differences with the neutrally-charged healthy cell membranes. Therefore, these chemical differences aid the electrostatic interaction of the positively-charged peptide and the negatively-charged tumor cell membranes (Dobrzynska et al., 2005; Hoskin and Ramamoorthy, 2008). The membranolytic mechanism was first discovered in a study of magainin and its synthetic analogs against hematopoietic and solid tumors (Cruciani et al., 1991). Cationic antitumor peptides might also induce disruption of intracellular targets by so-called non-membranolytic mechanisms. For instance, there are reports of antitumor peptides that cause necrosis by triggering intracellular apoptotic pathways (e.g., by inactivating mitochondria; activating caspase cascade) (Mader et al., 2005; Chen et al., 2012; Paredes-Gamero et al., 2012). Cationic antitumor peptides can also impair the activity of proteins of the signal transduction pathways involved in oncogene activities (Sharma, 1992). Both membranolytic and non-membranolytic mechanisms are discussed in this review and are represented in Figure 1.

Cationic peptides that kill cancer cells are arranged into two groups: (1) AMPs which are effective against bacteria and cancer cells but not against normal mammalian cells such as cecropins from insects and magainins from amphibians and (2) AMPs which are cytotoxic for bacteria, cancer cells, and normal mammalian cells such as melittin, tachyplesin II, human neutrophil defensins, and insect defensins (Papo and Shai, 2005; Hoskin and Ramamoorthy, 2008; Schweizer, 2009). It is believed that the difference between both groups relay mainly on the difference of the membrane of tumorous and normal cells (Hoskin and Ramamoorthy, 2008), therefore it would be essential to detailed understand these important changes at the molecular level. To date, it is here understood that besides the difference on the electrostatic charge between tumorous and normal cells cited above, the membrane fluidity may also interfere on the susceptibility of the peptide to disrupt the cells (Hoskin and Ramamoorthy, 2008). The fluidity of cancer cells is greater than that of mammalian normal cells (Sok et al., 1999) which may facilitate membrane destabilization favoring the binding of cationic antitumor peptides, and the amount of cholesterol, the major component of eukaryotic cell membranes (Simons and Ikonen, 2000), alters the membrane fluidity, therefore protecting eukaryotic cells from cytolytic effects of antitumor peptides. For instance, membrane-insertion of the antitumor peptide cecropin is shown to reduced when synthetic lipid vesicles and Gram-negative bacteria have their cholesterol amount enhanced (Silvestro et al., 1999). Furthermore, the surface area of the membrane of tumorous cells, where the number of microvilli is higher than on non-tumorous cells (Chaudhary and Munshi, 1995), may also allow an increased number of peptides to bind to the membrane (Chan et al., 1998a,b).

In the following sections, cationic antitumor peptides from both groups which are derived from many sources including plants, vertebrates, and invertebrates will be presented (see Table 1).

### Cationic antitumor peptides isolated from plants

High quantities of natural compounds and molecules from plants have been intensely studied for the treatment of a diverse number of diseases including cancer. Reviewed here is a specific class of cyclic peptides originally found only in plants as well as non-cyclic peptides with antitumor properties.

*Clitoria ternatea* is a rich source of a specific class of peptides named cyclotides, composed of a cyclic backbone combined with a conserved six cystine knot (Craik and Malik, 2013). A study carried out by Sen et al. (2013) has isolated five cyclotides named CT2, CT4, CT7, CT10, and CT12 (with an addition or removal of a net charge from −1 to +2) from this plant species. They showed significant cytotoxicity against human lung cancer cells (A549) and demonstrated a reduction of 2- to 4-fold of the IC_50_ (half maximal inhibitory concentration) value of cyclotides when compared to a mitotic inhibitor used in cancer chemotherapy. Moreover, these peptides were less cytotoxic against A549/paclitaxel (a sub-linage of A594) than only A549, which demonstrates a possible use of chemo-sensitization for treating cancer (Sen et al., 2013).

Along with cyclic peptides, many active non-cyclic antitumor peptides have also been isolated from different plants. For instance, the promiscuous peptide *Cn*-AMP1, isolated and purified from coconut water (*Cocos nucifera*), was tested against CACO-2- human epithelial colorectal adenocarcinoma cells and showed a 13% reduction of cell viability (Silva et al., 2012). Additionally, the peptide lunasin, isolated from soybeans and other seeds, was capable of suppressing *in vitro* and *in vivo* chemical carcinogen-induced tumorigenesis (Hsieh et al., 2010). *St*AP1 and *St*AP3, isolated from the potato *Solanum tuberosum*, were shown to induce apoptosis in Jurkat T leukemia cells (Mendieta et al., 2010; Guevara et al., 2011). Other examples are the peptides Cr-ACP, isolated from *Cycas revoluta*, and its acetylated-modified Cr-AcACP1, both repressors of cell proliferation of human epidermoid cancer (Hep2), and colon carcinoma through the induction of cell cycle arrest at the G0–G1 phase of Hep2 cells (Mandal et al., 2012).

Among chlorophyllous organisms, few studies have been developed. One example is the polypeptide *Chlorella pyrenoidosa* antitumor polypeptide (CPAP) isolated from the unicellular green algae *Chlorella pyrenoidosa*. It has shown the highest inhibitory activity on human liver HepG2 cancer cells (49%). CPAP induces apoptosis and necrotic death of HepG2 cells via membrane shrinkage, condensation and fragmentation of nuclear chromatin as well as formation of black apoptotic bodies (Wang and Zhang, 2013).

### Cationic antitumor peptides isolated from invertebrates

Bioactive peptides isolated from insects present many different activities and have strong potential as therapeutic agents (Table 1) (Chernysh et al., 2002). Cecropins, alloferons, and melittins are examples of AMPs isolated from insects which function as antitumor molecules with applications for various kinds of tumor cells (Figure 1). The AMP cecropin was first isolated from the giant silk moth *Hyalophora cecropia* (Steiner et al., 1981). Cecropins shares their potential antitumor activity with structural analogs from other families of AMPs such as magainins and defensins (Papo and Shai, 2005; Lehmann et al., 2006). It has been shown that two cecropin B analogs, cecropin B1 (CB1) which possesses two amphipathic helices, and cecropin B3 (CB3) which has two hydrophobic helices, exhibit strong cytotoxic activity against a number of human leukemia cell lines and do not lyse normal fibroblasts or erythrocytes (Srisailam et al., 2001). Both analogs exhibit drastically different mechanisms of actions on anionic lipid vesicles and show the importance of the structure and sequence of a cationic antitumor peptide and its potency toward different cancer cells (Srisailam et al., 2001). It has been claimed that the discontinuous helical segments in the structure of CB3 do not favor helix—helix interactions which are crucial for pore formation; these mechanisms are likely to be adopted by CB1 where the continuous helical conformation interacts with cell-surface structures such as microvilli in cancer cells. In a different study, CB1 was a more powerful cytolytic agent than cecropin B against HL-60 human promyelocytic leukemia cells (Chan et al., 1998a,b). Cecropin A and B have been shown to reduce the viability of bladder cancer cells (Suttmann et al., 2008) and to directly induce tumor cell lysis via cell membrane disruption, which stimulates cytolysis/necrosis. Furthermore, it was shown that these peptides are capable of imposing the disruption of mitochondrial membranes, consequently leading to the activation of apoptosis pathways (Suttmann et al., 2008).

Another important AMP isolated from insects is alloferon: a tridecapeptide isolated from the bacteria-challenged larvae of the blow fly *Calliphora vicina*. Synthetic alloferon I has been shown to act on tumor growth control in two different ways. It stimulates natural killer (NK) lymphocytes and interferon (IFN) *in vitro* by using mouse spleen lymphocytes and human blood mononuclear cells. The peptide was administrated in picomolar concentrations where its potential to stimulate natural cytotoxicity in these models was confirmed. Alloferon also induces IFN synthesis *in vivo* which was demonstrated using animal and human models, and consequently enhancing its antitumor activity (Figure 1). Based on these results, the researchers have suggested an interaction of alloferon anticancer activity with its immunomodulatory properties. The interferonogenic activity was more evident *in vitro* using human cells than *in vivo*, while in comparison to mouse cells the opposite result was observed. The NK-IFN network is well-documented and demonstrates the potential immune modulatory properties of this AMP. Furthermore, it has been recently shown that the combination of chemotherapy and alloferon I, referred to as pulse immune chemotherapy, demonstrated significant advantages compared to each treatment applied separately (Chernysh et al., 2002, 2012).

Also from the group of insects, the AMP melittin, isolated from *Apis mellifera*, is also an active molecule against antitumor cells. It is cytotoxic against human hepatocellular carcinoma (Tosteson et al., 1985; Wang et al., 2009a). Studies indicate that melittin damages cell membranes either via the barrel-stave mechanism, i.e., it acts under its membranolytic properties (Sui et al., 1994), or via its non-membranolytic properties through a mechanism that engages the hyperactivation of phospholipase A2 and the influx of Ca^2+^, resulting in the destruction of the transformed cells (Figure 1) (Sharma, 1992, 1993).

Another antiviral AMP within the group of invertebrates is the peptide AGAP isolated from the scorpion *Buthus martensii*. It has been reported to possess both analgesic and antitumor activities. Recently, a heterologous expression system has been constructed using small ubiquitin-related modifier-AGAP (SUMO-AGAP) which is a product of recombinant AGAP (rAGAP) linked with a hexa-histidine tag from *Escherichia coli*. This recombinant system showed considerable inhibition of lymphoma and glioma propagation (Gu et al., 2013). Using SW480 human colon cancer cells, it was proposed that rAGAP induces cell cycle arrest in the G0/G1 phase, attended by the decrease in the S phase without significant change in the G2/M phase (Gu et al., 2013).

Other sources of cationic peptides isolated from invertebrates are found in the marine ecosystem. The biodiversity of this environment has been shown to be a rich source of biologically active molecules and has been considered an unlimited resource of new antitumor agents (Zheng et al., 2011; Malaker and Ahmad, 2013). Several AMPs with antitumor activity have been isolated from marine invertebrates such as cyclic depsipeptide didemnins. Isolated from the ascidian of the genus *Trididemnum*, it has shown antitumor activity against L1210 leukemia cells *in vitro* and P388 leukemia and B16 *in vivo* (Rinehart et al., 1983). The antitumor role of the peptide kahalaide F from *Elysia rufescens*, a marine gastropod mollusk, has also been reported. It has shown significant *in vitro* and *in vivo* activity against non-small cell lung cancer, colon and human breast tumor cell lines, melanoma, androgen-independent prostate cancer and hepatocellular carcinoma (Martin-Algarra et al., 2009; Malaker and Ahmad, 2013). This peptide acts on the liposome membrane of tumor cells and modifies its basal function (Figure 1) (Hamann et al., 1996; Singh et al., 2008). Moreover, it modifies the role of the lysosomal membrane, leading to intracellular acidification and cell death, a characteristic that discriminates it from all other known antitumor agents (Gracia et al., 2006). This peptide also appears to inhibit the expression of certain specific genes that are involved in DNA replication and cell proliferation, thereby inhibiting tumor spreading and growth. Recently another marine AMP isolated from the bivalve *Meretrix meretrix*, named mere15, has shown to significantly inhibit the growth of human lung adenocarcinoma A549 xenograft in nude mice (Wang et al., 2012).

### Cationic antitumor peptides isolated from vertebrates

Many peptides have been discovered from a variety of vertebrates that are responsible for improving the innate immune response (Table 1) They have been found at relatively low concentrations in the normal tissues of mammals and are usually present within the granules of neutrophils, in mucosal or skin secretions from epithelial cells, and as the degradation products of proteins (Boman, 1995; Hancock, 2001). Among the mammalian organisms, bovines have been a promising source of molecules which show a broad range of physiological activities, including immune function enhancement and defense against pathogenic bacteria and viruses. Among these molecules, three AMPs named BMAP-27, BMAP-28 and lactoferricin have been reported as active antitumor peptides. The bovine myeloid AMPs BMAP-27 and BMAP-28 (27 and 28 amino acid residues, respectively) have shown cytotoxic activity against neoplastic cells. These peptides, when tested against fresh tumor leukocytes from patients affected by myeloid or lymphoid leukemia, have shown to increase membrane permeabilization and the influx of Ca^2+^, followed by DNA fragmentation, which is characteristic of programmed cell death (Figure 1) (Risso et al., 1998).

The cytotoxic activity of bovine lactoferricin (LfcinB) has been demonstrated *in vitro* with different rat and human cancer cell lines including leukemia, fibrosarcoma, various carcinoma, and neuroblastoma cells, and did not influence the viability of normal fibroblasts, lymphocytes, epithelial cells, endothelial cells, or erythrocytes (Yoo et al., 1997; Mader et al., 2005; Eliassen et al., 2006). It has been proposed that Lfcin targets tumor cells by the changes that occur in their cell membranes, such as the exposure of negatively-charged head-groups derived from the loss of phospholipid asymmetry in diseased cells (Gifford et al., 2005; Pepe et al., 2013). The activities against fibrosarcoma and neuroblastoma rat cells and human T-leukemia cells can be described by a mechanism that induces the formation of transmembrane pores allowing the peptide to enter the cytoplasmic compartment of the cancer cell, co-localize with negatively-charged mitochondria and consequently depolarize them, resulting in cytochrome C release or activation of the caspase cascade, thereby leading to cell death via apoptosis (Figure 1) (Mader et al., 2005; Pepe et al., 2013). Moreover, it may interfere with the interaction between growth factors and their receptors on the surface of endothelial cells, resulting in decreased endothelial cell proliferation and diminished angiogenesis (Mader et al., 2006). As reviewed by Gifford and colleagues, other mechanisms have been proposed. In brief, upon binding to the tumor cells, Lfcin is thought to trigger a Ca^2+^/Mg^2+^ endonuclease and oxidant-dependent apoptotic pathway. Although the structural parameters that describe the antitumor effects of Lfcin are very similar to those that describe its antibacterial activity, a higher net positive charge (+7 when compared to +4 for antibacterial activity) is required for antitumor activity to promote a strong electrostatic interaction between the peptide and the membrane (Figure 1) (Gifford et al., 2005).

Among the human-derived mammalian AMPs, the amphipathic α-helical LL-37 has been extensively studied as an antibacterial peptide. Besides its strong activity against bacteria, it has also shown to be cytotoxic against ovarian cancer (Chuang et al., 2009) and is toxic to eukaryotic cells at a slightly higher concentration (25–30 mM) (Hoskin and Ramamoorthy, 2008). Regarding its mechanism of action, it is known that upon binding to membranes it changes the head group conformation of phospholipids, induces positive curvature strain on lipid bilayers, and significantly disorders the hydrophobic core of the membranes, exhibiting the carpet-like rather than the channel/pore-forming mechanism of cytotoxicity (Figure 1) (Henzler Wildman et al., 2003; Henzler-Wildman et al., 2004; Hoskin and Ramamoorthy, 2008).

Within the class of amphibians, the order Anura has been shown to be a rich source of AMPs. Their skin has diverse physiological activities and forms an essential part of their defense systems. In response to a multiplicity of stimuli, AMPs can be secreted from specific glands onto the dorsal surface and into the gut of the amphibian (Bevins and Zasloff, 1990; Barra and Simmaco, 1995; Doyle et al., 2003).

In comparison to synthetic magainins A, B, and G, the natural peptide magainin-2, isolated from the skin of the frog *Xenopus laevis*, is the most efficient peptide and causes fast lysis of hematopoietic and solid tumor cell lines including many human bladder cell lines (Cruciani et al., 1991; Jacob and Zasloff, 1994). Magainin-2 has been shown to enter the cell membrane of HeLa human cervical carcinoma cells by binding numerous magainin helices forming a toroidal pore in the lipid molecules of artificial membranes (Matsuzaki et al., 1996). It has also been reported that pore formation is followed by a dispersing membrane potential and leakage of intracellular molecules, consequently leading to cell death (Takeshima et al., 2003). Magainin are shown to access the cytosolic compartment of cancer cells and cause the mitochondrial pathway of apoptosis via a mechanism that involves cytochrome c release into cytoplasm and an amplified proteasome activity, confirming its apoptotic effect (all these mechanisms are shown in Figure 1) (Westerhoff et al., 1989; Cruz-Chamorro et al., 2006).

Another example of potential antitumor AMPs belonging to the order Anura are gaegurin and buforin. The peptide gaegurin was purified from the skin of the Korean frog *Rana rugosa*. It is a potent mediator of cytolysis using either the carpet or barrel-stave mechanism (Park et al., 1994; Hoskin and Ramamoorthy, 2008) and is also reported to follow the mitochondria pathway of apoptosis as described above for the magainin mechanism of action (Li et al., 2000; Mai et al., 2001).

Buforin II, a linear α-helical peptide similar to cecropins and magainins, was isolated from the stomach of the *Bufo bufo gargarizans* and is known to penetrate cell membranes through non-permeabilizing pore-like structures which allow its translocation into the cytoplasm without cell lysis, and consequently inhibits intracellular functions (Park et al., 1998). It has been shown that the cytotoxic activity of buforin II is low when compared with citropin 1.1, pexiganan MSI-78 and protegrin 1, and that it does not permanently disrupt the cell membrane like other molecules, i.e., its antitumor activity corroborates with the mechanism described above (Koszalka et al., 2011). A synthetic analog of buforin II named buforin IIb has shown greater cytolytic activity against cancer cells (leukemia, breast cancer, non-small cell lung cancer, CNS cancer, melanoma, renal, ovarian, prostate, and colon cancer) than buforin II (Lee et al., 2008).

From the group of marine vertebrates, the cationic peptide pardaxin isolated from the small fish *Pardachirus marmoratus* has been shown to be a potential active antitumor peptide against human sarcoma (Huang et al., 2011). It has been reported that this peptide disrupts the membrane via the barrel-stave mechanism (Hallock et al., 2002), and changes its transmembrane orientation depending on membrane composition (Hoskin and Ramamoorthy, 2008).

### Cationic antivirus peptides

**Figure 2:**
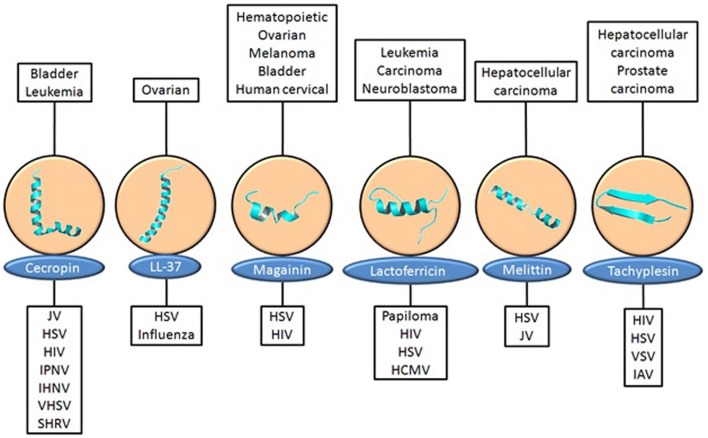
**Promiscuous cationic peptides with antitumor and antiviral activities**. Each promiscuous peptide and their various tumors against which present cytotoxic activity **(top)** and viruses against which present antiviral activity **(bottom)**. #PDB from left to right, 2IGR, 2K6O, 2LSA, 1Z6V, 2MLT, 1WO1. HCMV, human cytomegalovirus; HIV, Human immunodeficiency virus; HSV, herpes simplex virus; IAV, influenza; IHNV, infectious hematopoietic necrosis virus; IPNV, infectious pancreatic necrosis virus; Jv, Junin virus; SHRV, snakehead rhabdovirus; VHSV, viral hemorrhagic septicemia; VSV, vesicular stomatitis virus.

The success of viruses in evolution has been assured by four general attributes: genetic variation, variety in means of transmission, efficient replication within host cells, and the ability to persist in the host (Wagner et al., 1999). Due to these attributes, the control of viral diseases has not been an easy task. Despite the existence of antiviral drugs, there is a need to explore novel antiviral compounds in order to control emerging viral pathogens. In this perspective, AMPs are an alternative in drug design. Several cationic antiviral peptides from various sources have been isolated since the 1980s (Table 2) and they have shown strong potential for novel therapeutic drugs against many viral infections. Due to the promiscuity of these peptides, it is possible to verify a broad spectrum of antiviral activities within the same peptide. Moreover, this promiscuous activity can be extended to simultaneous cytotoxic activity against tumor cells (Figure 2). The first study reporting an antiviral role of a cationic peptide was published in 1986, in which the activity of α-defensin was described as inhibiting a number of viruses including herpes simplex virus types 1 and 2 (HSV), cytomegalovirus (CMV) as well as inhibiting the vesicular stomatitis virus with human neutrophil peptide 1 (HNP1) *in vitro* (Daher et al., 1986; Findlay et al., 2013). Since then, many reports have shown the antiviral activity of cationic host-defense peptides such as α-, β-, and θ-defensins, and the use of effective antiviral therapy with cathelicidins, as previously reviewed (Findlay et al., 2013). It is very promising that in the last years many new antiviral peptides have been either identified or synthesized in order to aid the development of new therapeutic antivirus therapies.

**Table 2:** **Cationic antiviral peptides from different sources, their application, and their mechanisms of action**.

**Peptide**	**Source (s)**	**Group**	**Application**	**Mechanism of action**	**References**
**HUMAN HEALTH**					
Alloferon 1	*Calliphora vicina*	Insect	IAV	Immunomodulatory activity	Chernysh et al., 2002
Alloferon 2					
Brevinin-1	*Rana brevipoda*	Frog	HSV	Viral inactivation	Yasin et al., 2000
Caerin 1.1 Caerin 1.9 Maculatin	–	Amphibian Skin	HIV	Disrupts the integrity of the virion membrane	Vancompernolle et al., 2005
CAP37	*Homo sapiens*	Human leococytes	HSV-1	Disrupts the envelope and/or capsid	Gordon et al., 2009
			AdV		
Cecropin	*Hyalophora cecropia*	Insect	JV	Suppresses viral protein synthesis Cellular target Suppresses viral gene expression	Wachinger et al., 1998; Albiol Matanic and Castilla, 2004
			HSV		
			HIV		
Circulin A	*Chassalia parvifolia*	Plant	HIV		Daly et al., 1999
Defensin	*Homo sapiens*	Human	HSV	Interacts with glycosaminoglycans Inactivates viral particle Cellular target Unknown	Daher et al., 1986; Nakashima et al., 1993; Gropp et al., 1999; Yasin et al., 2000; Bastian and Schafer, 2001; Sinha et al., 2003
			IAV		
			HCMV		
			VSV		
			HIV		
			AdV		
Dermaseptin	Genus *Phyllomedusa*	Frog	HIV	Disruptis viral membrane	Belaid et al., 2002
			HSV		
Didemnins A	Genus *Trididemnum*	Tunicate	HSV	Inhibits RNA and DNA viral replication	Rinehart et al., 1981; Aneiros and Garateix, 2004
Didemnins B			Parainfluenza		
			Dengue virus		
HNP-1	*Homo sapiens*	Human	HSV	Blocks early steps of viral replication	Ganz et al., 1985; Bastian and Schafer, 2001; Hook et al., 2006
HNP-3			AdV		
Hp1090	*Heterometrus petersii*	Scorpion	HCV	Disrupts viral membrane integrity	Yan et al., 2011
Indolicidin	*Bos taurus*	Bovine	HIV	Inhibits integrase	Robinson et al., 1998
			HSV	Targets viral glycosaminoglycans	
Lactoferricin	*Homo sapiens*	Human, Bovine	HCMV	Activity at virus-cell interface	Andersen et al., 2001; Jenssen et al., 2004; Mistry et al., 2007
			HIV		
	*Bos taurus*		HSV	Blocks heparan sulfate	
			Papilloma		
LL-37	*Homo sapiens*	Human	HSV	Viral receptor-based mechanisms	Yasin et al., 2000; Barlow et al., 2011
			IAV		
Magainin	*Xenopus laevis*	Frog	HSV	Suppresses viral gene expression	Aboudy et al., 1994; Albiol Matanic and Castilla, 2004
			HIV		
Mellitin	*Apis mellifera*	Insect	HSV	Cellular target	Wachinger et al., 1998; Yasin et al., 2000; Albiol Matanic and Castilla, 2004
			JV		
Microspinosamide	*Sidonops microspinosa*	Marine sponge	HIV	Inhibits cytopathic effect of HIV-1 infection	Rashid et al., 2001
Pa-MAP	*Pleuronectes americanus*	Fish	HSV	Interacs with viral envelope	Migliolo et al., 2012
PAP	*Phytolacca americana*	Plant	HIV	Inhibits viral protein synthesis	Kaur et al., 2011
			HBV		
			HSV		
Polyphemusin	*Tachypleu tridentatus*	Horseshoe crab	HIV	Binds gp120 and CD4	Nakashima et al., 1992; Tamamura et al., 1996
Protegrin	*Homo sapiens*	Human	HIV	Unknown	Yasin et al., 2000; Steinstraesser et al., 2005
			HSV	Viral inactivation	
Tachyplesin	*Tachypleus tridentatus*	*Horseshoe crab*	HIV	Virus-cell fusion	Morimoto et al., 1991; Murakami et al., 1991; Yasin et al., 2000
			HSV	Viral inactivation	
			VSV	Viral envelope	
			IAV		
θ-defensin	*Homo sapiens*	Human	HIV	Binds glycosylated gp120	Cole et al., 2002; Yasin et al., 2004
			HSV	Binds gB and blocks viral attachment	
**ANIMAL HEALTH**					
Cecropin B	*Hyalophora cecropia*	Insect	IHNV	Disrupts the viral envelope	Chiou et al., 2002
CF17		Synthetic	VHSV	Disintegrates the viral capsids	
			SHRV		
			IPNV		
Epinecidin-1	*Oreochromis*	Fish	NNV	Agglutinates NNV virions into clump	Chia et al., 2010
TH 1-5	*mossambicus*	Shrimp			
cSALF	*Penaeus monodon*				
Pleurocidin MDPle	*Limanda limanda*	Fish	VHSV	Disrupts the viral membrane via toroidal pore formation model	Falco et al., 2009
**AGRICULTURE**					
Potide-G	*Solanum tuberosum L*	Plant	PVYO	Unknown	Tripathi et al., 2006
PAP	*Phytolacca americana*	Plant	TMV	Inhibit viral protein synthesis	Chen et al., 1991
			CMV		
			CaMV		
Indolicidin	*Bos taurus*	Bovine neutrophils	TMV	Unknown	Bhargava et al., 2007
Peptamine	*Pseudomonas chlororaphis O6*	Bacteria	TMV	Unknown	Park et al., 2012
Analogs of melittin	*Apis mellifera*	Synthetic	TMV	Cellular target	Marcos et al., 1995

Adapted from (Jenssen et al., 2006); –Not reported. AdV, adenovirus; CaMV, cauliflower mosaic virus; CMV, cytomegalovirus; HBV, hepatitis B virus; HCMV, human cytomegalovirus; HCV, hepatitis C virus; HIV, Human immunodeficiency virus; HSV, hermpes simplex virus; IAV, influenza; IHNV, infectious haematopoietic necrosis virus; IPNV, infectious pancreatic necrosis virus; JV, junin virus; NNV, nervous necrosis virus; PVYO, potato virus YO; SHRV, snakehead rhabdovirus; TMV, tobacco mosaic virus; VHSV, viral hemorrhagic septicemia; VSV, vesicular stomatitis virus; VSV, vesicular stomatitis virus.

### Cationic antiviral peptides drugs applied to human health

**Figure 3:**
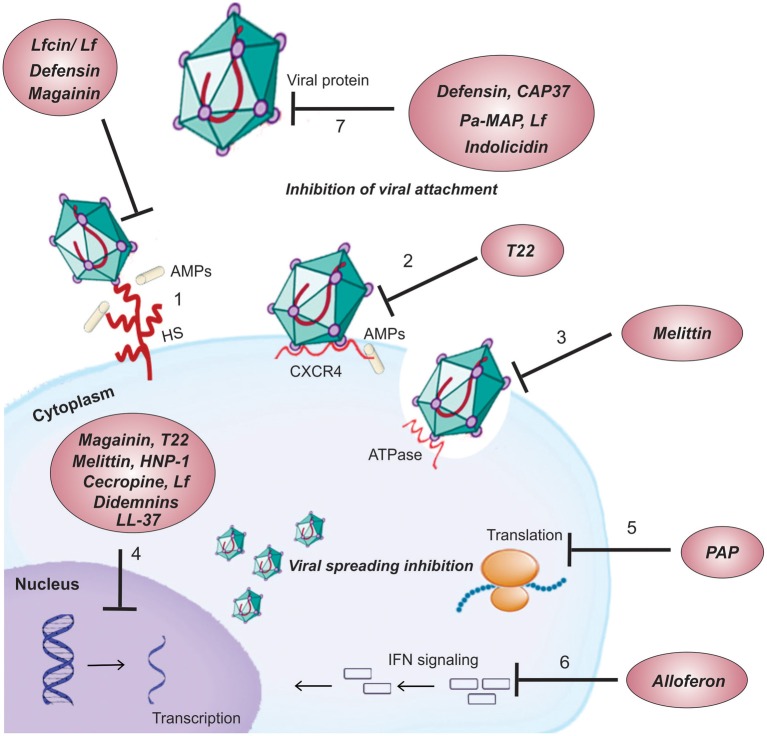
**Mechanisms of action of cationic antiviral peptides**. *Cell surface targets*: (1) Interaction of peptides with different glycosaminoglycan (e.g., HS) present on the cell surface competing with the virus for cellular binding sites. (2) Blocking of viral entry into the cell by binding the peptide to viral CXCR4co-receptor required for its entry. (3) Suppression of cell fusion by interfering with the activity of ATPase protein. *Intracellular targets*: (4) Suppression viral gene expression. (5) Inhibition of peptide chain elongation by inactivating the ribosome. (6) Activation of an immune modulatory pathway by induction of NK and IFN. *Viral protein targets*: (7) Binding of peptides to viral proteins causing inhibition of adsorption/virus-cell fusion.

Cationic antiviral peptides have been isolated from various sources and present broad antiviral activities against several viruses with different antiviral mechanisms of action (Table 2). They can either inhibit viral attachment by binding to viral targets on the host cell surface, or target viral proteins, therefore blocking viral fusion and entry into the host cell. Another mechanism of action is intracellularly driven where spreading of the virus is inhibited through the suppression of viral gene expression, inhibition of translation or by immune modulatory activities (Figure 3).

### Cationic antiviral peptides isolated from invertebrates

AMPs isolated from the group of invertebrates which present strong antiviral activity against maladies that affect human health are well-represented by peptides such as melittin, cecropin, and alloferon. Described above as an antitumor peptide, melittin has also been reported to have inhibitory activity against enveloped viruses such as HIV-1, HSV-2, and the Junin virus (JV), an arenavirus, including this peptide under the concept of promiscuity (Figure 2). A proposed mechanism of action has suggested that melittin suppresses cell fusion mediated by HSV-1 syncytial mutants probably by interfering with the activity of the Na^+^ K^+^ ATPase, a cellular enzyme involved in the membrane fusion process (Albiol Matanic and Castilla, 2004). Analysis of the effect of melittin on the production of HIV-1 transcripts was assayed in acutely infected T-cells cultured with various concentrations of melittin. Levels of all HIV-1 transcript classes were suppressed (reduction of ~30% when compared with cells without melittin) in a dose-dependent manner (Wachinger et al., 1998). Another mechanism whereby melittin interferes with viral gene expression has been proposed and involves intracellular immunization against HIV (Figure 3). Melittin interfere in the process of cellular signal transduction, such as the activation of phospholipase A2 for instance, and the decrease in activities of calmodulin and protein kinase C (Sharma, 1993; Fisher et al., 1994; Gravitt et al., 1994). These properties may therefore change the balance and activities of cellular stimulators of HIV transcription (as NFkB, AP-1 and NFAT) or induce inhibitory factors (interferon-induced cellular inhibitor) (Wachinger et al., 1998).

The peptides alloferon 1 and 2 have shown activity against the influenza virus, through the same mechanism described above for its role as an antitumor peptide, by induction of immune modulatory activities. Both activities against tumor and viral infections emphasize the mechanisms of cell-mediated natural cytotoxicity and IFN synthesis (Figure 3). Therefore, these peptides seem to be potential candidates as biopharmaceutical compounds containing the capability of improving important effector mechanisms of the innate immune response (Chernysh et al., 2002).

Another example of invertebrate peptides with antivirus activity against human virus-related diseases is the synthetic peptide T22 ([Tyr5, 12, Lys7]-polyphemusin II), which is associated with the promiscuous peptide tachyplesin (Figure 2) and the peptide polyphemusin, which are abundant in the hemocytes of the horseshoe crab *Tachypleus tridentatus* and *Limulus polyphemus*, respectively. It has shown potent antiviral activity against HIV-1 and HIV-2 *in vitro*. The inhibitory activity of this peptide is related to its specific binding to a chemokine receptor CXCR4, which serves as a co-receptor for the entry of HIV-1 into T cells (Figure 3) (Nakashima et al., 1992; Tamamura et al., 1998).

### Cationic antiviral peptides isolated from vertebrates

There are several AMPs derived from mammalian sources which have demonstrated strong activity against many viruses that compromise human health (Table 2). In this group are included defensins, Lfcin and LL-37 (Figure 2). Defensins are potent candidates for the development of antiviral drugs. They are peptides with conserved structures, usually with α-helix and antiparallel β-sheets stabilized by disulfide bonds (Terras et al., 1992; Bastian and Schafer, 2001; Franco et al., 2006), and belong to three subfamilies, designated α, β, and θ defensins. The HNP-1 is an α-defensin that have been widely tested for its antiviral activity. This peptide has been reported to deactivate HSV-1 and HSV-2, CMV, vesicular stomatitis virus, influenza virus and human respiratory adenovirus type-5 (AdV-5) (Daher et al., 1986; Yasin et al., 2004). Treatment with this peptide has shown a decrease of adenoviral infection by more than 95% in 293 cells infected with AdV-5) (Bastian and Schafer, 2001). θ-defensins are circular octadecapeptides with two antiparallel β-sheets that are bridged by a tri-disulfide ladder and connected by two β-turns (Yasin et al., 2004). This class of peptide has been reported to be miniature lectins that bind to the protein gp120 of human immunodeficiency virus type 1 (HIV-1) with high affinity, blocking its entry into the host cell (Figure 3) (Munk et al., 2003).

The promiscuous peptide LfcinB (Figure 2) has shown inhibition against many viruses such as HIV-1, HSV-1, and HSV-2, human cytomegalovirus (HCMV), respiratory syncytial virus, hepatitis B and C viruses (HBV and HCV, respectively), adenovirus, and rotavirus (Van Der Strate et al., 2001; Andersen et al., 2003). The activity of Lfcin has been claimed to be attributed to the affinity of this peptide for carbohydrates, which are viral binding sites on the cell membrane, such as heparin sulfate (HS) and glycosaminoglycans (GAGs), thereby blocking viral entry (Andersen et al., 2003). The antiviral activity of LfcinB and LfcinH (human lactoferricin) against HSV has been verified with the ability of this peptide to interact with HS and block viral entry. It has been found that the positive net charges of the peptides are critical for affinity with HS which is due to the many negatively-charged sulfate groups present in the molecule (Jenssen et al., 2004). Together with Lfcin, the peptides human α-defensin, LL-37 and magainin have also been reported to bind to GAGs in order to perform their respective activities (Figure 3) (Jenssen et al., 2006). It has also been shown that a stabilized secondary structure is important for antiviral activity for both LfcinB and LfcinH (human lactoferricin). The higher potency of LfcinB against some viruses compared to LfcinH is attributed to the β-sheet conformation of LfcinB in solution compared with the α-helical structures of LfcinH (Jenssen et al., 2004).

Lactoferrin (Lf), the protein from which the peptide Lfcin is derived, shows antiviral activity against a number of viruses as much as seven times greater than that of Lfcin, proposing that either the size of the molecule is important or that other regions of LF contribute to the antiviral activity (Andersen et al., 2003; Gifford et al., 2005). The positive charge of Lf was found to be important for antiviral activity against human HCMV (Valenti and Antonini, 2005). When negatively-charged groups were added to Lf by succinylation, the antiviral potency was mostly decreased, whereas the addition of positive charges to Lf through amination of the protein resulted in increased anti-HCMV activity (Harmsen et al., 1995). On the other hand, when tested against HIV-1, a 4-fold stronger antiviral effect of Lf was observed when negatively-charged groups were added (Harmsen et al., 1995). The proposed mechanism of action for anti-HIV activity was that Lf and the charged-modified protein bind strongly to the V3 loop of the gp120 envelope protein, increasing the net negative electric charge of viral particles, and resulting in inhibition of virus-cell fusion and entry of the virus into cells (Puddu et al., 1998) Another report of direct interaction of viral proteins with Lf has been observed in HCV. Inhibition of virus-cell adsorption was verified in human hepatocytes PH5CH8 when bLf was mixed prior to the viral infection in serum containing HCV, and no antiviral activity of bLf was observed after internalization of HCV in human hepatocytes (Ikeda et al., 1998). This activity has been reported to be involved in the ability of hLF and bLF to bind to E1 and E2 proteins of the viral envelope, indicating that neutralization occurs in order to prevent the adsorption of HCV in the hepatocytes (Yi et al., 1997). During *in vivo* experiments, bLF protected the host mice against infection of the mouse cytomegalovirus (MCMV) when the peptide was injected prior to the viral infection, but failed to protect the mice when the injection was performed after MCMV infection (Shimizu et al., 1996).

Another human-derived peptide which targets envelope/membrane is the promiscuous peptide CAP37, first isolated from the granule fractions of human PMNs. It has been shown to have potent activity against viruses (HSV-1 and Adenovirus), bacteria (*Pseudomonas aeruginosa, E. coli, Salmonella typhimurium, Staphylococcus aureus, and Enterococcus faecalis*), and fungi (*Candida albicans*). Its structure/function differs from the others peptides here mentioned. It is known that cystine residues forming intramolecular disulfide bridges are necessary for the antibacterial function of CAP37 but are not required for its antiviral activity, which has been suggested to be involved in the rupture of the envelope and/or capsid (Gordon et al., 2009).

The promiscuous peptide LL-37 (Figure 2) differs from the above defensins, Lfcin, and bLf in its mechanism of action. Instead of targeting cell surface molecules, it inhibits viral spreading by inactivating intracellular targets (Figure 3). LL-37 have nuclear localization signals and have been related to interact with DNA, directly influencing viral nucleic acid synthesis (Sandgren et al., 2004). The same is applied to indolicidin, a peptide isolated from the cytoplasmic granules of bovine neutrophils (Hsu et al., 2005). However, besides its DNA-binding ability, it has also shown activity against HIV and HSV through a membrane-mediated antiviral mechanism (Robinson et al., 1998).

Marine organisms are also included as a source of cationic antiviral peptides (Table 2). One example is the promiscuous peptide Pa-MAP isolated from the fish *Pleuronectes americanus*, which beyond having shown broad antimicrobial activity against bacteria (*E. coli and S. aureus*), fungi (*Candida parapsilosis, Trichophyton mentagrophytes, and Trichophyton rubrum*) and tumor cells in culture (CACO-2, MCF-7, and HCT-116), also has activity against viruses (HSV-1 and HSV-2). The antiviral mechanism of this peptide has been suggested to involve its interaction with the viral envelope (Migliolo et al., 2012; Teixeira et al., 2013).

A group of cationic antiviral peptides from vertebrate and invertebrate sources has been tested by Carriel-Gomes et al. (2007). This study has shown *in vitro* evaluation of the cytotoxicity and antiviral activity of nine AMPs against many human viruses (Carriel-Gomes et al., 2007). They were PW-2 recombinant, tachyplesin-1 from limulid, gomesin from spider, clavanin A from tunicate, magainin from frog, synthetic HCTF, penaeidin-3 and ALF from shrimp, and mytilin A from mussel. These peptides have different structures, origins and antiviral activities against HSV-1, AdV-5, and rotavirus SA11 (RV-SA11). All evaluated peptides were cytotoxic and had antiviral activities in different degrees. The peptides PW-2, ALF and penaeidin-3 exhibited higher antiviral activity against HSV-1. The peptides ALF and clavanin A showed significant antiviral activity against AdV-5 with clavanin A exhibiting a greater inhibition of viral replication (Carriel-Gomes et al., 2007).

### Antiviral peptides drugs applied to animal health

Several studies have reported the efficiency of AMPs active against viruses that cause diseases in humans. However, few studies have demonstrated the potential application of these peptides in animal health. Here we present studies showing the application of antiviral peptides in aquaculture. Viral diseases have emerged as the most serious infectious problems for the fish aquacultural industry, and studies have found promising cationic antivirus peptides being used against several viruses including rhabdoviruses such as viral hemorrhagic septicemia virus (VHSV) and infectious hematopoietic necrosis virus (IHNV), which are responsible for the greatest losses in aquaculture production.

The native cecropine B and its synthetic analog CF17, have demonstrated activity against major viral fish pathogens such as infectious hematopoietic necrosis virus (IHNV), viral hemorrhagic septicemia virus (VHSV), snakehead rhabdovirus (SHRV) and infectious pancreatic necrosis virus (IPNV). The mechanism of action involved in the inhibition of viral replication by peptides is related to the direct disruption of the viral envelop and the disintegration of the viral capsids (Chiou et al., 2002).

Chia and collaborators reported that both peptides tilapia hepcidin 1-5 (TH 1-5) and cyclic shrimp anti-lipopolysaccharide factor (cSALF) exhibited noticeable antiviral activity *in vitro* against nervous necrosis virus (NNV), a virus that has caused mass mortality of numerous marine fish species at their larval stage. The antiviral mechanism of both peptides was by agglutinating NNV virions into clump and preventing viral entry into the cells (Chia et al., 2010). Together with hepcidin 1-5, the peptide hapcidin 1 has also shown antiviral activity *in vivo* against NNV in the Japanese rice fish medaka (*Oryzias latipes*). Pre-treatment, co-treatment or post-treatment with epinecidin-1 or hepcidin 1-5 has shown to be effective in promoting a significant increase in medaka survival when infected with NNV (Wang et al., 2010a).

Falco and collaborators (2009) have reviewed the role of many AMPs as antiviral agents in the fish farm industry. Among these molecules there are pleurocidin and the HNP1. Pleurocidin MDPle, isolated from the Mud dab fish (*Limanda limanda*), has presented antiviral properties against VHSV. It is likely that it disrupts the viral membrane under the same mechanisms adopted for its antibacterial activity (Falco et al., 2009). The HNP1 is a defensin that has demonstrated antiviral effects against the same virus as MDPle. It has been claimed that, as belonging to the class of defensins, HNP1 can deactivate the enveloped virus by interacting with GAGs present on the viral surface, altering the ability of these glycoproteins to bind to their receptors at the target cells. Moreover, this peptide has also been shown to stimulate the immune modulatory system of infected fish cells by modulating IFN-related mechanisms (Falco et al., 2007, 2009).

### Antiviral peptide drugs applied to agriculture

Cationic antivirus peptides may also be applied to plant protection against viruses that cause diseases in crops. Among the various symptoms of viral infections in plants, stunting, mosaic patterns, yellowing, leaf rolling, ring spot, necrosis, wilting, and other developmental abnormalities can be observed (Hull, 2002). These symptoms consequently cause a decrease in production leading to economic losses. Strategies to combat viral diseases in plants are usually directed to the prevention or reduction of infection which may have, however, adverse effects on human health and the environment. Therefore, AMPs have become promising alternatives for protecting crops against viral diseases while simultaneously protecting human health and the environment.

A cyclic peptide of 7 amino acid residues called peptamine from *Pseudomonas chlororaphis* O6 has shown antiviral activity against the tobacco mosaic virus (TMV). Antiviral bioassays of tobacco plants suppressed 95% of TMV disease. The mode of action by which peptamine suppresses the disease is still unknown, but it has been speculated that this peptide may induce systemic resistance against TMV (Park et al., 2012). The peptide PAP, a ribosome inactivating protein (RIP) isolated from *Phytolacca americana*, was highly effective in inhibiting the formation of local lesions caused by TMV on tobacco leaves (Taylor et al., 1994). RIPs are known to cause damage to ribosomes by removing adenine residues from 28S rRNA through an N-glycosidase activity, and the removal of this base prevents binding of the elongation factor 2 (EF-2), consequently stoping the synthesis of proteins (Kaur et al., 2011). Another cationic antiviral with activity against TMV is subK7I, a synthetic analog of melittin. This peptide has sequence and structural similarity to an essential domain of the TMV coat protein and was found to possess highly specific antiviral activity. Bioassays of tobacco leaves upon addition of the analog to the solution before inoculation of the virus has demonstrated a reduction of more than 90% of infectivity of TMV with dependent doses (Marcos et al., 1995).

Tripathi and colleagues isolated a small antiviral peptide of 5.57 kDa called potide-G from potato tubers resistant to Potato Virus Y (PVY). Results of real-time PCR showed that the application of 10 μg of purified potide-G was sufficient to reduce virus accumulation by 50% on average from the PVY infection in susceptible “Winter Valley” cultivars. The authors have reported that this peptide isolated from resistant potatoes offers new opportunities for the development of new biological pesticides against plant viruses (Tripathi et al., 2006).

## Production of cationic antitumor and antiviral peptides

While peptides have great potential for use in antitumor drugs, there are some limitations that need to be addressed. It is well-known that they can be used in a number of different ways in treating cancer such as vaccines, hormones, tumor targeting with cytotoxic drugs and radioisotopes, and anti-angiogenic peptides (Thundimadathil, 2012). Currently, there are about 60 approved peptide drugs on the market and it is expected to reach an estimated $12 billion USD in sales by the end of 2013 (Pichereau and Allary, 2005). All of these investments have resulted in more than 100 peptide-based drugs which are already available on the global market, representing about 1.5% of all drug sales (Lax, 2010; Craik et al., 2013). Out of four peptide drugs on the market which have reached global sales over $1 billion USD, three peptides are used in treating cancer directly or in the treatment of episodes associated with certain tumors: leuprolide acetate (Lupron; $2.12 billion), goserelin acetate (Zoladex; $1.14 billion), and octreotide acetate (Sandostatin; $1.12 billion) (Reichert et al., 2010; Thundimadathil, 2012). Mendoza and colleagues have summarized approximately 30 peptides which were discovered/developed as peptide-based anticancer drugs in order to provide foundations for therapies (Mendoza et al., 2005).

However, when it comes to the market of AMPs as antitumor drugs, the scenario is much different. Although over 1000 potential therapeutic AMPs have been isolated and characterized from different sources, only limited success has been achieved in clinical trials (Hu et al., 2011).

There are currently only around ten AMPs in either preclinical or clinical trial phases (Fox, 2013) and few of these present antitumor activity. Their major limitations are the poor bioavailability due to their instability and insolubility related to the intrinsic physicochemical properties, potential toxicity to host cells, tissue distribution, poor pharmacokinetic issues, and the cost of large scale production (Rotem and Mor, 2009; Hu et al., 2011). Despite these disadvantages, antitumor peptides have potential due to their high specificity and potency against malignant cells. Therefore, numerous studies have been performed in order to improve their bioavailability and reduce their cost of production. Much effort has been put into the mimicry of antitumor peptides to alter their features in order to achieve robustness and safety. These approaches have become important and promising for improving the therapeutic potential of antitumor peptides (Rotem and Mor, 2009).

HDP mimicry has been performed by constructing oligomers of acyl-lysyl and/or lysyl-acyl-lysyl (OAKs), which has turned out to be the first designed system to show antitumor potential *in vivo*. This system was able to translocate across the membrane and interact with multiple intracellular targets, including mitochondria, especially the inner membrane which contains a relatively high portion of negatively-charged phospholipids that might mediate interactions with these HDPs. Moreover, it was associated with an improved toxicity profile when compared to doxorubicin, an anthracycline antibiotic used in chemotherapy. It has also been observed that local administration of both OAK and doxorubicin resulted in a complete disappearance of tumors in 50% of treated mice, while in the remaining 50% tumors were minuscule, thereby suggesting that the synergic effect of this therapy was both potent and well tolerated. The OAK might also damage lysosome structure and/or interfere with the function of 50 hydrolases that normally process the cell's major macromolecules (Held-Kuznetsov et al., 2009).

In another study, sequence optimization and modification based on natural peptide sequences and traits was performed and it has shown that the system G(IIKK)nI-NH 2 (being *n* = 3 − 4) was effective against HeLa and HL60 cancer cell lines with 50% growth inhibition concentrations (Hu et al., 2011). Mai and colleagues have designed a novel antitumor peptide, DP1, derived from a synthetic AMP that significantly induces apoptosis in solid tumors by local injection. It was composed of a protein transduction domain (PTD), PTD-5, fused to the AMP KLAKLAK_2_, an antimicrobial apoptosis-inducing peptide that upon internalization causes mitochondrial swelling and disruption of the mitochondrial membrane leading to apoptosis (Mai et al., 2001; Thundimadathil, 2012).

Rational design of novel peptides has also been performed to create six analogs of temporin-1CEa, a naturally occurring α-helical and amphipathic AMP derived from skin secretions of the Chinese brown frog *Rana chensinensis* (Yang et al., 2013). These analogs were synthesized with either increased cationicity or increased/decreased hydrophobicity generally by substituting neutral and acidic amino acids with lysine or leucine residues on the polar face and non-polar face of the α-helix in order to evaluate the correlation between anticancer activity and physical properties of these peptides. All peptides showed potent anticancer activities against three cancer cell lines (MCF-7, Bcap-37, and MDA-MB-231). This study has suggested that the strategy of increasing the cationicity and keeping moderate hydrophobicity of naturally occurring AMPs is suitable to improve their cytotoxicity against tumor cells and decrease their hemolytic activity (Yang et al., 2013).

Synthetic links between two functional domains, an AMP (KLAKLAKKLAKLA K) and the isoDGR, (isoAsp-Gly-Arg), a derivative of a targeted delivery tool, was performed to construct a novel antitumor peptide. It was shown that this novel construction can selectively kill CD13^−^/α_*v*_β_3+_ breast cancer cells in both *in vitro* and *in vivo* experiments. The mechanism of action was claimed to inhibit angiogenesis by binding to α_*v*_β_3+_ which is up-regulated on tumor cells and tumor endothelial cells (Hou et al., 2013).

These studies strongly suggest that the peptidomimetics is a potent tool for developing new antitumor peptides. Moreover, these molecules may have their potential increased when it functions as an adjuvant therapy in conjunction with radiotherapy, chemotherapy, or surgical procedures.

When antiviral therapy is considered, the major barriers to the development and use of effective therapy are the current expensive approaches to impairing the completion of the viral growth cycle in the infected cell without being toxic to the surrounding normal cells, and diagnosing the viral disease before it is too late for effective therapy. It has also been claimed that the reason for the lack of progress in antiviral therapy is the selectivity, since the viruses are functionally incorporated into the host cells, and therefore it is difficult to select a proper tag (Kinchington et al., 1995; Abonyi et al., 2009). Furthermore, a relevant barrier is applied to the market of antivirus drugs, which concentrate efforts on a few viruses. It is known that out of 60 antiviral drugs that have thus far been approved by the US Food and Drug Administration (FDA), almost half of them target HIV-1 with the remaining half used for the treatment of HBV and HCV infections, HSV, CMV, varicella-zoster virus (VZV), and influenza (IAV) (De Clerq, 2008; Findlay et al., 2013). There are currently 15 peptide-based strategies against viruses in different stages of clinical trials as candidates for therapeutic drugs against many viruses (Thakur et al., 2012). However, none are cationic antiviral peptides, although reports of their high activity are exciting enough to include them as potential candidates, as is the case of alloferon. It has been shown that preventive and/or therapeutic administration of alloferon essentially increased the survival rate and suppressed virus reproduction in mice intra-cerebrally infected with HSV-2. Alloferon-based therapy of HSV, HBV, and HCV infections is now under extensive preclinical study (Chernysh et al., 2002).

Synthetic analogs of several naturally occurring AMPs have been made in attempt to identify important structural features contributing to their antiviral activity as well as to optimize these molecules in order to develop the shortest, cheapest, most stable and functional molecules at lower concentrations (Jenssen et al., 2006). Recently, the first antivirus peptide prediction method, based on the collected peptides which were experimentally proven for antiviral activity, was developed and named AVPpred (Thakur et al., 2012). This method is specific for antivirus peptides, where 25 physicochemical properties to develop antivirus peptide-physico models, amino acid composition, and sequence alignment implementing BLASTP algorithms for prediction of AVPs were used.

### Heterologous production of antitumor and antiviral cationic peptides

Beyond the use of peptide mimicry to develop synthetic peptides, the advance in the use of heterologous systems for production of cationic peptides has been a promising alternative. Many groups have been performing recombinant technology in order to optimize the production yield of cationic peptides using animal cells (Brocal et al., 2006), yeast (Wang et al., 2009b), plant (Lee et al., 2011), and bacteria systems (Wang et al., 2011).

For instance, the expression and purification of the recombinant human α-defensin 5 has been performed in *Pichia pastoris*, and it has been reported that this peptide was functionally expressed and it showed *in vitro* activity to block human papillomavirus infection (Wang et al., 2009b). The housefly cecropin peptide has also been expressed in this organism, however, neither antiviral nor antitumor activities have been tested in this specific system (Jin et al., 2006). The lack of both activity could also be observed for the expression of the peptide pleurocidin, which has been expressed in both yeast (Burrowes et al., 2005) and fish cell lines (Brocal et al., 2006), although only in the latter case antibacterial activity was tested.

The concept of plant-made biopharmaceuticals has also been lately explored for the expression of antiviral cationic peptides. For example, the peptide retrocyclin, an important AMP which can be used as therapeutic agent against HIV-1 viral infections, have been expressed and characterized in tobacco chloroplasts genome, and its antiviral activity was confirmed against TMV infection (Lee et al., 2011). The peptide cecropin was also expressed in both camelina (*Camelina sativa*) (Zakharchenko et al., 2013) and tomato (*Solanum lycopersicum*) (Jan et al., 2010). Nevertheless, in these studies the tests performed did not include antiviral or antitumor activities.

The most common organism for construction of heterologous expression systems is bacteria, and *E. coli* is the most used (Parachin et al., 2012). This bacterium has been used for expression of cecropin (Liang et al., 2006), lactoferricin (Luo et al., 2007), human α and β defensins (Wang et al., 2010b), buforin (Wang et al., 2011), indolicin (Morin et al., 2006), and LL-37 (Moon et al., 2006). Two different systems for expression of cecropin, one fused to enterokinase (Xu et al., 2007a) and other hybrid system fused to ubiquitin (Xu et al., 2007b) were constructed, and both systems were active against Gram-positive and negative bacteria, and fungi. Another two hybrid systems have also been constructed to actively express the peptide lactoferricin (Kim et al., 2006; Feng et al., 2011). Furthermore, many bioactive human defensin have been successfully expressed in *E. coli* such as the human α-defensin 6, which showed to inhibit HSV-2 infection (Wang et al., 2010b), the β-defensin 5 and 6 (Huang et al., 2008), the β-defensin 26 and 27 (Huang et al., 2009), β-defensin 2 (Zhong et al., 2006), and β-defensin 4 (Xu et al., 2006; Li et al., 2010).

As shown above, many studies have shown that the heterologous expression of cationic peptides is feasible, and in many cases, depending on their size and structure, is a better alternative for production of pharmaceuticals than the synthetic synthesis (Parachin et al., 2012). However, none of the studies cited above have optimized the large-scale production of the peptides expressed. Doubtless it is important to mention that many efforts have been made to efficiently develop or improve the cost-effective methodology (e.g., new expression systems and optimized fermentation processes) for production of AMPs, as well as to overcome barriers involving both recombinant technology processes and the economic scenario of biotechnology and pharmaceutical companies in order to change the drug-development current scenario.

### Nanoformulation as drug-delivery system

In order to overcome the restraints to the use of cationic peptides described above, nanoformulation techniques have emerged as a potent biological tool to improve the delivery and stability and of these molecules.

The primary goals of drug delivery for cancer therapy is to improve the therapeutic index of anticancer drugs by increasing the amount of drug delivered to the tumor site and decreasing its exposure to healthy tissues (McDaniel et al., 2010). Several microencapsulation technologies have been developed for use in the pharmaceutical industry, such as hydrogels, liposomes, nanoemulsions, and nanoparticles (Onwulata, 2012). Therefore, the development and improvement of nanoparticles have shown great promise to overcome the delivery barriers of pharmaceuticals (Zhang et al., 2012).

Recently Wang and Zhang (2013) encapsulated a CPAP, a dose-dependent antiproliferation peptide and inducer of the post-G1 cell cycle arrest in gastric cancer cells. Two methods were used: complex coacervation (edible alginate, CaCl2 and chitosan involved) to perform a microencapsulation of CPAP, and ionotropic gelation (edible chitosan and sodium tripolyphosphate involved) to perform a nanoencapsulation process. The former refers to the phase separation of a liquid precipitate/phase when solutions of two hydrophilic colloids are mixed under suitable conditions, and the latter is based on the ability of polyelectrolyte counter ions to cross link to form hydrogels (Wang and Zhang, 2013). Their results demonstrated that the encapsulation efficiency of microencapsulation (74.5%) is much greater than that of nanoencapsulation (30.1%), but their polypeptides contents are similar (12.7 vs. 12.3%). The *in vitro* release tests revealed that CPAP was well-preserved against gastric enzymatic degradation after micro/nanoencapsulation and the slowly-controlled release in the intestine could be achieved. Although further *in vivo* studies are required to verify these findings, this study has provided a basis for the development of encapsulated antitumor peptides (Wang and Zhang, 2013).

Another study has performed encapsulation of an antitumor peptide in order to develop an effective gene-modified tumor cell vaccine. In this study, the gene of interleukin-27 (IL-27), a novel IL-6/IL-12 family cytokine, was transfected in LL/2 (Lewis lung cancer cell) by using a cationic liposome. This resultant tumor cell vaccine then containing both tumor associated antigen (TAA) of LL/2 cells and secreted mIL-27 (mouseIL-27) at a relative high level, could induce protective antitumor immunity in mice, which was claimed to be an attribute of high-cytokine production achieved by the liposome-DNA complex. It was also observed that LL/2-mIL-27 cell vaccine up-regulated IFN-γ in serum and improved local CD4+ and CD8+ T cell infiltration in mice. This study shows an important and promising strategy for AMP-based therapeutic approach against tumors (Zhang et al., 2013b).

Regarding cationic antiviral peptides, a nanoformulation technique has also been studied as a potent bio-tool to improve their delivery and stability. Nanoformulation of the amphiphatic α-helical peptide p41, a positively-charged analog of C5A peptide derived from the HCV protein, was performed to treat HIV/HCV co-infection. The cationic antiviral peptide was incorporated into anionic poly (amino acid)-based block copolymers prepared via eletrostetic coupling. The nanocomplexes were ca. 35 nm in size, stable at physiological pH and ionic strength. The *in vitro* antiviral activity against both HCV and HIV was retained and their intrinsic cytotoxicity was attenuated. The *in vivo* APN were able to decrease the viral load in mice transplanted with human lymphocytes and HIV-1-infected. Overall, these findings indicate the potential of these formulations for stabilization and delivery of antiviral peptides while maintaining their functional activity (Zhang et al., 2013a).

## Concluding remarks

Many natural cationic peptides from the immense biodiversity of several groups of organisms have been isolated and have demonstrated great potential as antiviral and antitumor agents. Furthermore, the development of novel synthetic analogs of these natural molecules has been an important biological tool for enhancing their activities and facilitating the screening process for new natural AMPs. Studies on antitumor and/or antiviral AMPs have demonstrated that these molecules work as excellent therapeutic agents and even lead to greater success when combined with traditional treatments. The number of antitumor and/or antiviral AMPs has been increasing and is expected to lead to a change in their current scenario, from their entry into clinical trial to their availability on the market. A crucial step for this change to occur is to continue researching and identifying their mechanisms of action and to discover new targets, followed by studies on developing new potential delivery systems.

### Conflict of interest statement

The authors declare that the research was conducted in the absence of any commercial or financial relationships that could be construed as a potential conflict of interest.
